# A Study on the Inflammatory Response of the Brain in Neurosyphilis

**DOI:** 10.1002/advs.202406971

**Published:** 2024-11-22

**Authors:** Qiyu Zhang, Jie Ma, Jia Zhou, Hanlin Zhang, Mansheng Li, Huizi Gong, Yujie Wang, Heyi Zheng, Jun Li, Ling Leng

**Affiliations:** ^1^ Stem cell and Regenerative Medicine Lab Institute of Clinical Medicine State Key Laboratory of Complex Severe and Rare Diseases Peking Union Medical College Hospital Chinese Academy of Medical Sciences and Peking Union Medical College Beijing 100730 China; ^2^ Department of Dermatology Institute of Clinical Medicine State Key Laboratory of Complex Severe and Rare Diseases Peking Union Medical College Hospital Chinese Academy of Medical Sciences and Peking Union Medical College National Clinical Research Center for Dermatologic and Immunologic Diseases Beijing 100730 China; ^3^ State Key Laboratory of Medical Proteomics Beijing Proteome Research Center National Center for Protein Sciences (Beijing) Beijing Institute of Lifeomics Beijing 102206 China

**Keywords:** blood‒brain barrier, inflammation‐mediated brain injury, neurosyphilis, proteomics, single‐cell transcriptomics

## Abstract

Neurosyphilis (NS) is a clinical condition caused by infection of the central nervous system (CNS) by *Treponema pallidum* (*Tp*) that can lead to asymptomatic meningitis and more serious neurological diseases, such as dementia and blindness. However, current studies on the pathogenesis of NS are limited. Here, through the integration analysis of proteomics and single‐cell transcriptomics, Toll‐like/NF‐κB signaling is identified as the key pathway involved in CNS damage caused by *Tp*. Moreover, monocyte‐derived macrophages are key cells involved in the inflammatory response to *Tp* in the CNS of NS patients. In addition, it is found that inflammatory cells in peripheral blood may cause neurological damage through disruption of the blood‒brain barrier (BBB) in individuals with NS. Notably, activation of the Toll‐like/NF‐κB signaling pathway, as well as dysregulation of neural function, is likewise validated in an in vitro NS brain organoid model. In conclusion, the results revealed the mechanisms of inflammation‐mediated brain injury in *Tp*‐induced NS and provided new ideas for the clinical treatment of *Tp* infection.

## Graphical abstract

This study reveals the mechanisms of inflammation and immune‐mediated brain injury in patients with Neurosyphilis (NS). By proteomic analysis of brain tissues and single‐cell sequencing of cerebrospinal fluid from NS patients, researchers identified Toll‐like/NF‐κB signaling as the key pathway involved in the damage of central nervous system in NS. Meanwhile, researchers verified the damaging effects of peripheral inflammatory cells on the brain using organoids.

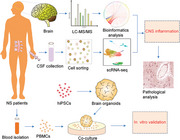

## 1 Introduction

The incidence of syphilis has been on the rise since the 21st century.^[^ ^1^ ^]^ In the early stages of infection, *Treponema pallidum* (*Tp*) can invade distant tissues, including tissues of the central nervous system (CNS), causing abnormal cerebrospinal fluid (CSF) findings in up to 50% of patients.^[^ ^1^ ^]^ *Tp* infection of the CNS in early syphilis patients can cause potentially devastating neurological complications years later if left untreated.^[^ ^2^ ^]^ Thus, timely diagnosis and treatment of neurosyphilis (NS) are crucial. Early NS is usually characterized by asymptomatic meningitis, which may manifest only as an abnormal cell compositions in the CSF.^[^ ^3^ ^]^ Other manifestations of NS include general paresis, tabes, the meningovascular syphilis, gummatous syphilis, and epilepsy, which are often atypical depending on the disease course and type of lesions.^[^ ^4^ ^]^ Due to variable clinical features and the lack of gold standard tests, the diagnosis of NS is difficult.^[^ ^5^ ^]^ Therefore, an in‐depth investigation of the pathogenesis of NS will be useful for the early diagnosis of NS and the development of treatment strategies.

*Tp* can evade the host immune system and cross the blood‐brain barrier (BBB) into the CNS in the early stages of syphilis.^[^ ^6^ ^]^ Notably, the organism traverses BBB through various pathways that enhance endothelial permeability and modulate the expression of tight junction proteins.^[^ ^7^ ^]^ Several studies have reported that the development of NS is closely related to abnormal immune responses and inflammation.^[^ ^8^, ^9^, ^10^, ^11^ ^]^ *Tp* promotes microglial apoptosis and inhibits their migration, effectively evading immune clearance mechanisms.^[^ ^12^, ^13^ ^]^ The immune response triggered by *Tp* within the CNS leads to localized inflammatory reactions, with the subsequent release of cytokines playing critical roles in the pathogenesis of NS.^[^ ^14^ ^]^ Elevated levels of pro‐inflammatory cytokines, such as IL‐6 and IL‐8, in cerebrospinal fluid correlate with increased BBB permeability.^[^ ^15^ ^]^ Specific *Tp* proteins, including TP0751, interact with endothelial cells, altering their biological characteristics and facilitating the pathogen's invasive potential.^[^ ^16^ ^]^ Previously, we characterized CSF specimens from NS patients using proteomic techniques and found a large enrichment of proteins associated with immune and inflammatory responses.^[^ ^17^ ^]^ However, a comprehensive understanding of the molecular features of the *Tp*‐infected CNS is still lacking. A major impediment to the study of the pathogenesis of syphilis is the lack of stable disease models.^[^ ^18^ ^]^ Preclinical models commonly used in syphilis research include in vitro coculture models with cottontail rabbit epithelial cells, rabbit models, macaque models, and mouse models.^[^ ^19^ ^]^ There is a lack of robust in vitro human models for syphilis research, and the information gathered from the animal models described above is limited due to species differences.

In recent years, human stem cell‐derived organoid models have shown great potential for studying disease pathogenesis and drug screening.^[^ ^20^, ^21^, ^22^, ^23^, ^24^ ^]^ Several studies have established brain organoids to model viral infections and neuroinflammation.^[^ ^25^, ^26^, ^27^ ^]^ There are no reports on the use of organoid models to study the pathogenesis of syphilis. In this study, through further proteomic analysis of brain tissues from NS patients, single‐cell RNA sequencing (scRNA‐seq) of CSF samples from NS patients, and construction of a brain organoid inflammation‐induced injury model, we elucidated the mechanisms of inflammatory and immune damage in CNS injury caused by *Tp*.

## 2 Results

### 2.1 Integration of Proteomics and Single‐Cell Transcriptome Analysis Reveals Molecular Characteristics of the Inflammatory Response in the Brain Tissues of NS Patients

**Figure 1:**
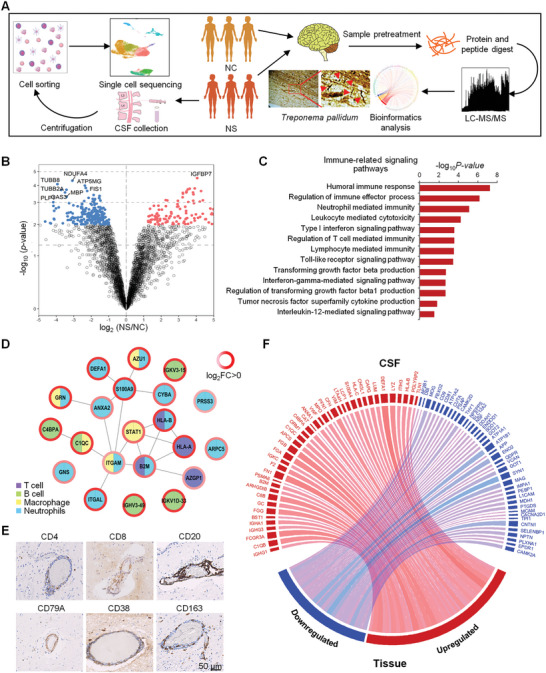
Proteome characteristics of brain tissues from NS patients. A) Schematic of the methods used for quantitative proteomics, scRNA‐seq, and bioinformatics analysis of brain tissues and CSF from NS patients. B) Volcano plots showing the upregulated and downregulated proteins expressed in the brain tissues of patients with NS (n = 3) compared to those of control individuals (NC, n = 5). Proteins outside the significance threshold are in red (−log_10_ (p value) > 2 and log_2_ (NS/NC) > 1, upregulated) or blue (−log_10_ (p value) > 2 and log_2_ (NS/NC) < −1, downregulated). Differential expression analysis is performed using a moderated *t* test implemented in the R package limma and the proteins with a Benjamini–Hochberg (BH) adjusted *p* value ≤ 0.01 indicate statistical significance. C) Immune‐related signaling pathway analysis of the upregulated proteins in the brain tissues between the NS versus control groups. The bars represent the ‐log_10_ p value of enrichment analysis. Upregulated proteins are those with BH adjusted *p*‐value < 0.01 and log_2_ (NS/NC) > 1. D) Interaction network of proteins that are differentially expressed in brain tissues between the NS and control groups. A map of the immune system‐associated categories is labeled with different colors. The log_2_ NS/NC protein abundance ratio was used to measure the differential protein expression, and it was mapped on the rings with the red‒blue color palette, with deep blue indicating much lower than normal expression rates, pale colors indicating near‐normal expression rates, and deep red indicating much higher than normal expression rates. E) Immunohistochemical analysis of CD4, CD8, CD20, CD79A, CD38, and CD163 around the blood vessels of brain tissues from NS patients (scale bar: 50 µm). F) Circos plot showing the co‐expression correlation of the upregulated (red font) or downregulated (blue font) proteins of the NS versus control groups in CSF and brain tissue samples. The upregulated and downregulated proteins are those with BH adjusted *p*‐value < 0.01 and log_2_ (NS/NC) > 1 or log_2_ (NS/NC) < −1, respectively. The line width indicates the absolute log2 (NS/NC) values of the differentially expressed proteins.

To investigate the molecular pathological features of *Tp* infection, we collected brain tissue samples from patients infected with *Tp* for quantitative proteomic analysis (**Figure** **1**A). A total of 115 upregulated and 219 downregulated proteins were identified in the brain tissues of patients in the NS group compared with the control group (Figure 1B; Tables S1 and S2, Supporting Information). The upregulated proteins were mainly involved in the humoral immune response (C4BPA, IGHV3‐49, IGKV1D‐33, and IGKV3‐15), neutrophil‐mediated immunity (DEFA1, S100A9, CYBA, ARPC5, and PRSS3), T‐cell‐mediated immunity (HLA‐A, HLA‐B, B2M, and AZGP1), and macrophage‐mediated immunity (STAT1, AZU1, GRN, C1QC, and ITGAM) (Figure 1C‐D), indicating that there were inflammatory cells in the brain tissues of NS patients. To verify the proteome results, we immunostained the brain tissue samples and found many CD4+ and CD8+ T cells, CD20+ and CD79A+ B cells, CD38+ plasma cells, and CD163+ macrophages near the blood vessels of the brain tissue (Figure 1E).

**Figure 2:**
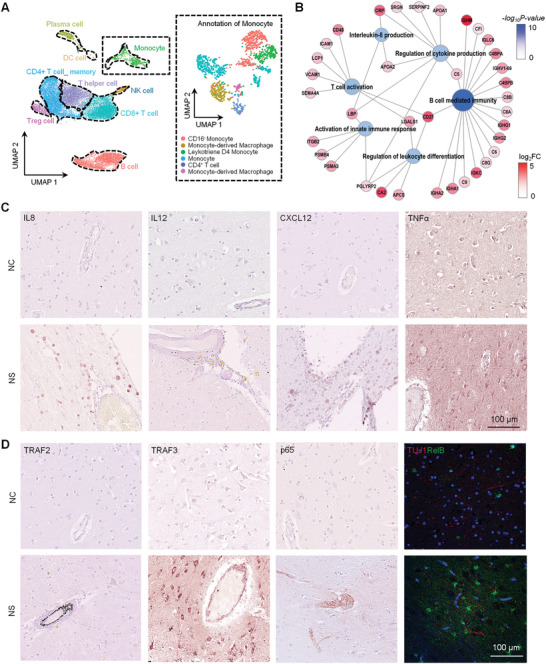
Single‐cell transcriptomic characteristics of CSF from NS patients. A) Uniform manifold approximation and projection (UMAP) plots of the scRNA‐seq data of the CSF from NS patients (n = 3). A total of 20 327 cells were represented. The dotted box represents the subset annotation of monocytes. The major cell groups detected form the CSF samples were manually annotated by referencing their corresponding markers and color‐coded. B) Interaction network showing protein expression profiles associated with B‐cell‐mediated immunity, T‐cell activation, cytokine production, leukocyte differentiation, the innate immune response, and interleukin‐8 production in the NS versus control groups. The blue circles represent the pathways enriched in the upregulated proteins in the NS versus control comparison. The blue circle size corresponds to the number of proteins in the pathway and the color gradient represents the ‐log_10_ p value of functional enrichment analysis. Red circles indicate the highly and weakly enriched proteins according to log2‐fold changes in the NS versus control groups. C) Immunohistochemical analysis of IL8, IL12, CXCL12, and TNFα in the brain tissues of the NS and control groups (scale bar: 100 µm). D) Immunohistochemical staining of TRAF2, TRAF3, and p65, and immunofluorescence staining of TUJ1 and RelB in the brain tissues of the NS and control groups (scale bar: 100 µm).

Through correlation analysis with the previously published data, the upregulated and downregulated proteins in the brain tissue of NS patients were similar as those identified in CSF samples from the NS patients (Figure 1F). For example, proteins involved in T‐cell‐mediated immunity (B2M and HLA‐B), B‐cell‐mediated immunity (IGHG1, C1QB, IGHG3, IGHA1, C8B, IGKC, APCS, C1QC, and C4BPA), and neutrophil‐mediated immunity (BST1, F2, ORM1, CAT, MPO, LTA4H, HLA‐C, CHI3L1, DEFA1, and LYZ) were both upregulated in the brain tissue and CSF samples from NS patients (Figure 1F). Therefore, we collected CSF from three NS patients for single‐cell transcriptome analysis. Most of the immune cells were T cells, including CD4+ T cell memory (24.98%), CD8+ T cells (22.44%), T helper cells (18.79%), and regulatory T (Treg) cells (3.67%). B cells (19.02%) also accounted for a considerable proportion. Additionally, there were monocytes (5.88%), plasma cells (2.75%), natural killer (NK) cells (2.28%), and dendritic cells (DCs) (0.20%), in the CSF of NS patients (**Figure** **2**A; Figure S1 and Table S3, Supporting Information), which was consistent with the proteomic and histopathological results of brain tissues. These results indicated that inflammatory cell infiltration is the main pathological feature in brain tissues of NS patients.

### 2.2 *Tp* Infection Activates the Toll‐Like/NF‐κB Signaling Pathway in the Brain Tissues of NS Patients

To further investigate the pathogenic mechanism of brain tissue damage caused by inflammatory cells, we analyzed the functions of the differentially expressed proteins between the brain tissues of NS patients and control individuals. The results showed that various inflammatory‐related pathways, such as those associated with IL‐1, IL‐12, Toll‐like receptors (TLR), TGFβ, IFNγ, and TNFα, were activated in the brain tissues of NS patients (Figure 1C). Additionally, proteins that are associated with cytokine production, such as those related to the production of IL‐8 (LBP, APOA2, and CRP), were detected in the CSF of NS patients (Figure 2B). Furthermore, histological staining revealed that the levels of IL8, IL12, CXCL12, TNFα, IL6, IL1β, IFNα, and BAFF were increased in the brain tissues of NS patients compared to control individuals (Figure 2C; Figure S2A, Supporting Information). Next, to investigate the mechanisms of these inflammatory responses, we focused on the enriched TLR signaling pathway identified by the proteomic analysis of brain tissues of NS patients (Figure 1C). We found that the TLR4 and CD14 and the downstream NF‐κB signaling‐related proteins TRAF6, TRAF3, and TRAF2 of the TLR signaling pathway were upregulated in the brain tissue of NS patients compared to control individuals (Figure 2D; Figure S2A, Supporting Information). In addition, we also observed upregulated expression and nuclear translocation of canonical (p65) and noncanonical (RelB) NF‐κB transcription factors in brain tissues of NS patients (Figure 2D; Figure S2E, Supporting Information). These findings suggested that TLR4/NF‐κB signaling plays an important role in initiating inflammation in brain tissues of NS patients. These inflammatory factors can induce proinflammatory and chemotactic effects through the canonical NF‐κB pathway and promote B‐cell development and survival, lymphocyte adhesion, and T‐cell costimulation via the noncanonical NF‐κB pathway (Figure S2E).

In addition, we also detected high expression levels of proteins associated with B cell, T cell, leukocyte, and innate immune cell functions in the CSF of NS patients (Figure 2B), suggesting the occurrence of NS‐related brain inflammation. Notably, the monocytes had the greatest impact on cell biological processes (Figure S2B, Supporting Information). We further analyzed monocyte‐derived macrophages (Figure 2A), and found the genes enriched in macrophages of the CSF from NS patients were mainly enriched in the inflammatory response, including IL‐6, IFN‐1, TNF, IFNγ, and chemokine signals related genes (Figure S2C, Supporting Information), which is consistent with the previous results in the brain tissues of NS patients (Figure 2C; Figure S2A, Supporting Information). Taken together, these results indicated that inflammatory cells, mainly mononuclear cells/macrophages, damage the brain through the Toll‐like/NF‐κB signaling pathway during *Tp* infection.

### 2.3 Inflammatory Cells Cause Brain Dysfunction through Destruction of the BBB

**Figure 3:**
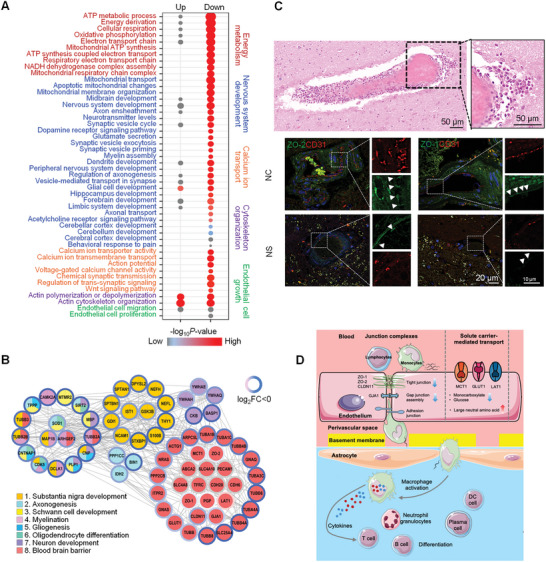
Dysregulated proteomic expression profiles in brain tissues from NS patients. A) The enriched biological processes of dysregulated proteins in the brain tissues of patients in the NS group (n = 3) compared to the control group (n = 5). The functional categories are shown on the right of the bubble diagram. The biological processes of downregulated proteins were analyzed using Gene Ontology (GO) enrichment analysis. Circles of different sizes represent the percentage of proteins included in each biological process. Red circles in the gradient indicate the degree of enrichment based on the p value of enrichment analysis. B) Interaction network of proteins that are differentially expressed in brain tissues between the NS (n = 3) and control (n = 5) groups. A map of the nervous system‐associated categories is labeled with different colors. The log2 (NS/NC) protein abundance ratio was used to measure the differential protein expression and was mapped on the rings with the red‒blue color palette, with deep blue indicating much lower than normal expression rates, pale colors indicating near‐normal expression rates, and deep red indicating much higher than normal expression rates. The gray rings represent the proteins that were either not identified in either group or the fold change values were not available. The gray solid lines represent interactions among the proteins in the brain tissues. C) Pathological analysis of inflammatory cell infiltration into brain tissue and the levels of tight junction proteins (ZO‐1 and ZO‐2) between endothelial cells and an endothelial cell marker (CD31) in the BBB (scale bar: 50, 20, and 10 µm). D) Mechanistic diagram of immune cells mediating nerve cell injury through damaging the BBB. The red and blue arrows represent the biological processes or substances in which the upregulated and downregulated proteins are located, respectively.

Next, to investigate how inflammation affects brain function in NS patients, we analyzed the dysregulated proteins in brain tissues of NS patients. We found that energy metabolism‐, nervous system development‐, calcium transport‐, cytoskeleton organization‐, and endothelial cell growth‐associated proteins were obviously downregulated (**Figure** **3**A). In particular, proteins associated with nervous system‐related functions, including substantia nigra development (NEFL, NEFH, THY1, S100B, etc.), axogenesis (BIN1, PPP1CC, IDH2, etc.), Schwann cell development (MTMR2, MBP, etc.), myelination (CAMK2A, TUBB2A, MAP1B, etc.), gliogenesis (TPPP, CNP, etc.), oligodendrocyte differentiation (SOD1, CNTNAP1, etc.), and neuronal development (BASP1, CKB, YWHAQ, etc.) were obviously downregulated (Figure 3B).

Our results demonstrated the infiltration of various types of inflammatory cells in brain tissues of NS patients (Figure 1E). Thus, we investigated the mechanism through which inflammatory cells enter the brain in response to *Tp* infection. We found that there were many inflammatory cells around blood vessels in the brain tissues of NS patients, and inflammatory cells infiltrated the brain tissue (Figure 3C), indicating that the inflammatory cells might have penetrated the BBB. When the inflammatory response is triggered, infiltrating leukocytes disrupt the interactions of several junctional adhesion molecules (JAMs) on endothelial cells and then penetrate the endothelial basement membrane via interactions between leukocyte‐ and endothelial cell‐expressed adhesion molecules.^[^ ^28^ ^]^ In addition, infiltrating leukocytes secrete proinflammatory cytokines, chemokines, and proteases, which damage endothelial cells; inflammatory cells can then attach to endothelial cells and secrete proteases such as matrix metalloproteinases, enabling them to cross the BBB and enter brain tissue from blood.^[^ ^29^ ^]^ Our results showed that BBB‐associated proteins including tight junctions (ZO‐1, and ZO‐2), gap junctions, and solute carriers (SLC25A4, SLC2A1, MCT1, and GLUT1) were decreased in brain tissues of NS patients (Figure 3B‐D; Figure S2D, Supporting Information), indicating that inflammatory cells in the peripheral blood entered the brain in response to *Tp* infection by penetrating the BBB.

### 2.4 The Mechanism of Inflammation‐Induced Damage in Brain Tissues of NS Patients was Verified in an In Vitro Brain Organoid Model

**Figure 4:**
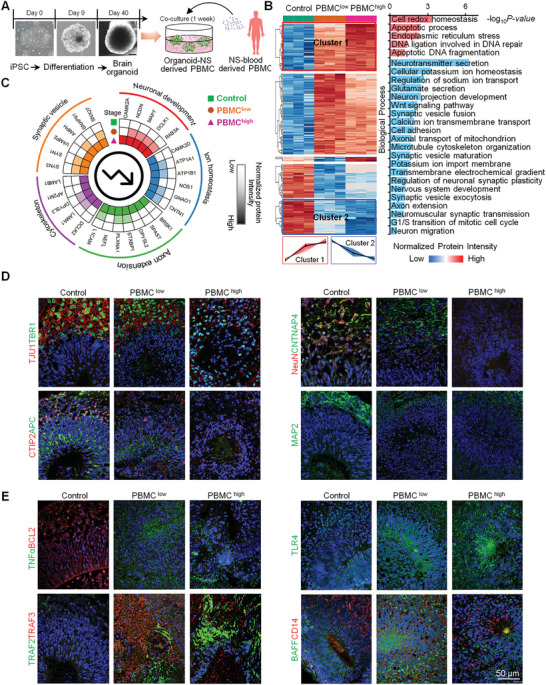
Proteomic characteristics of brain organoids cocultured with PBMCs from NS patients. A) A schematic overview of hiPSC‐brain organoid formation, coculture of organoids, and NS‐derived PBMCs in vitro. The bright field image represents brain organoid culture on days 0, 9, and 40 (scale bar: 200 µm). B) Heatmap showing the differentially expressed proteins (335 total proteins) in brain organoids among control (n = 3), PBMC^low^ (n = 3), and PBMC^high^ (n = 3) samples. Differential expression analysis is performed using a moderated *t* test implemented in the R package limma. And the proteins with a BH adjusted *p* value ≤ 0.01 in both the PBMC^low^ versus Control and PBMC^high^ versus Control indicate statistical significance. The proteins with log_2_ (PBMC^low^/Control) > 0 and log_2_ (PBMC^high^/PBMC^low^) > 0 are identified as gradually increased, while the proteins with log_2_ (PBMC^low^/Control) < 0 and log_2_ (PBMC^high^/PBMC^low^) < 0 are identified as gradually decreased. The red and blue empty frames or clusters 1 and 2 boxes represent the proteins whose expression gradually increased and decreased, respectively, from the control to PBMC^low^ and to PBMC^high^. PBMC^low^ and PBMC^high^ represent low and high concentrations of PBMCs, respectively, for brain organoid treatment. Histogram analysis showing the biological process of the proteins whose expression gradually increased (red) and decreased (blue) from the control group to PBMC^low^ and to the PBMC^high^ group, respectively. C) Brain function‐related proteins whose expression gradually decreased in the brain organoids treated with low or high concentrations of PBMCs. Each circle represents the concentration of PBMCs. Red, blue, green, purple, green, and orange squares correspond to the components or functions in which the different proteins are involved. Boxes in gradient color indicate the degree of enrichment based on normalized protein intensity performed by a moderated t‐test. D) Immunofluorescence analysis of functional brain markers (TUJ1, TBR1, CTIP2, APC, NeuN, CNTNAP4, and MAP2) in brain organoids treated with low and high concentrations of PBMCs (scale bar: 50 µm). E) Immunofluorescence analysis of inflammatory pathway‐associated proteins (TNFα, TRAF2, TRAF3, TLR4, BAFF, and CD14) and an antiapoptotic protein (BCL2) in brain organoids treated with low and high concentrations of PBMCs (scale bar: 50 µm).

To investigate the effect of inflammatory cell infiltration on the brain function of NS patients, we established an in vitro brain organoid culture system and cocultured it with different concentrations of peripheral blood mononuclear cells (PBMCs) isolated from blood of NS patients (Figure **4**A; Figure S3, Supporting Information). By proteomic analysis, a total of 3029, 3420, and 3469 proteins were identified in the control, PBMC^low^ concentration‐treated, and PBMC^high^ concentration‐treated samples, respectively (Figure S4A and Table S4, Supporting Information). Principal coordinate analysis (PCoA) revealed that the three groups were clearly distant from each other (Figure S4B, Supporting Information). In total, 304 and 328 differentially expressed proteins were identified between the control and PBMC^low^ or PBMC^high^ groups, respectively (Figure S4C, D and Table S4, Supporting Information). With the increase in PBMC concentration (low to high), 79 proteins were gradually upregulated in the brain organoid, which are involved mainly in cell redox homeostasis, apoptosis, DNA damage, and other related processes (Figure 4B). Additionally, 56 proteins were gradually downregulated, and these proteins are involved mainly in neurotransmitter secretion, ion homeostasis (CAMK2D, ATP1A1, ATP1B1, NOS1, GNAO1, and CNTN1), neuronal development (CAMK2A, NCDN, MAPT, DCLK1, and RAB3A), axon extension (BRSK1, SPAST, DPYSL2, STXBP1, PLXNA1, NEFL, and L1CAM), and synaptic vesicle transport (SYN3, SYN1, VAMP2, AMPH, SNAP91, and SNX27) (Figure 4B,C). In addition, proteins involved in signaling pathways related to brain development, such as the Wnt pathway and cell cycle, were downregulated significantly (Figure 4B), which was consistent with the findings in brain tissues of NS patients. Furthermore, through histological staining, we demonstrated that functional brain markers, including neuron‐specific markers (TUJ1, NeuN, MAP2, TBR1, CTIP2, and CNTNAP4), an astrocyte marker (GFAP), and an oligodendrocyte marker (APC), decreased significantly in the brain organoids in the PBMC^high^ and PBMC^low^ groups compared to the control group (Figure 4D; Figure S4E, Supporting Information), indicating that PBMCs from blood of NS patients seriously damaged the function of the brain organoids. In addition, we also found that TLR4/NFκB signaling‐associated proteins (TLR4, CD14, TRAF2, and TRAF3) and downstream inflammatory factors (TNFα and BAFF) were upregulated in the PBMC‐treated organoids (Figure 4E), which was consistent with the findings in brain tissues of NS patients. Moreover, immunofluorescence staining revealed that the expression of the antiapoptotic marker BCL2 in brain tissues of NS patients and PBMC‐treated organoids was downregulated (Figure S2A and Figure 4E, Supporting Information), indicating that inflammatory factors produced by immune cells may eventually lead to the apoptosis of brain nerve cells. Further, we verified that the expression levels of ITIH1, ITIH2, and ITIH4, which were upregulated in the CSF and brain tissues of NS patients and identified as potential diagnostic markers in the previous study.^[^ ^17^ ^]^ The results showed that the levels of the three proteins increased in the PBMC‐treated organoids (Figure S4E, Supporting Information), indicating that ITIHs upregulation may be a response to inflammation, which is consistent with the findings of the previous study. These results suggested that inflammatory cells in the blood of NS patients penetrate the BBB and damage brain tissue during *Tp* infection.

## 3 Discussion

*Tp* has been designated “the stealth pathogen” due to its early transmission and immune evasion capabilities.^[^ ^2^ ^]^ There are many obstacles to studying the pathogenesis of syphilis, such as the limitations of in vitro cultures and the lack of suitable experimental animal models.^[^ ^6^, ^30^ ^]^ In recent years, multiomics analyses, such as single‐cell and proteomic analyses, have provided new means to investigate intra‐ and intercellular processes under physiological and disease conditions at the molecular levels.^[^ ^31^ ^]^ In this study, based on a previous proteomic study of CSF of NS patients,^[^ ^17^ ^]^ we further performed proteomic analysis of *Tp*‐infected brain tissue to investigate the molecular pathological features of NS. Consistent with the observations in CSF, substantial activation of humoral and cellular immune responses and various perivascular inflammatory cells were observed in brain tissues of NS patients. To further investigate the inflammatory cell types in the nervous system, we performed scRNA‐seq analysis of CSF samples from NS patients. Multiple cell types, including T cells, B cells, plasma cells, NK cells, DCs, and macrophages, were identified. Among these cell types, T cells and B cells were the two largest populations, which is consistent with previous findings of studies on the CSF of NS patients.^[^ ^8^, ^32^ ^]^

A characteristic feature of patients with NS is the elevated levels of various inflammatory factors and chemokines in the CSF.^[^ ^10^, ^11^, ^33^ ^]^ Several previous studies have reported that recombinant *Tp* proteins can activate the TLR or NF‐κB signaling pathway to promote the production of proinflammatory cytokines, including IL‐6, IL‐8, and MMP9, in macrophages,^[^ ^34^ ^]^ keratinocytes,^[^ ^35^ ^]^ fibroblasts,^[^ ^36^ ^]^ and monocytes^[^ ^37^ ^]^ in 2D cell cultures. Although these studies revealed *Tp*‐induced inflammatory responses, these cellular models cannot mimic the microenvironment of real human organs. In our study, by analyzing inflammatory signals in brain tissues of NS patients we found that the TLR4/NF‐κB signaling pathway may be a key pathway mediating neurological damage caused by *Tp*, thus leading to severe neurovascular and metabolic dysfunction. In addition, among all of the cell types circulating in CSF of NS patients, monocyte‐derived macrophages are key cells involved in the inflammatory response to *Tp*, which is also related to the close interaction of macrophages with *Tp*.^[^ ^38^ ^]^ Notably, BBB‐related proteins were downregulated in NS patients, providing indirect evidence that *Tp* and inflammatory cells enter the brain through the BBB.^[^ ^7^, ^39^ ^]^

Since substantial activation of immune cells and inflammatory pathways in brain tissue and CSF is a key feature of NS, we performed proteomic analysis of brain organoids cocultured with PBMCs from patients with NS to further explore the injurious role of circulating inflammatory cells in the pathogenesis of NS. As expected, proteins related to inflammation and apoptosis were upregulated, whereas proteins related to normal neurodevelopment and neurotransmission were downregulated in the PBMC‐treated brain organoids. We also found that PBMCs promoted the activation of TLR4/NF‐κB inflammatory signaling in brain organoids, further supporting the mechanism of neurological damage in the brain tissues of NS patients. Although *Tp* can't produce lipopolysaccharides (LPS), the dendritic cell‐derived exosomes induced by *Tp* can stimulate endothelial cells via the TLR4/MyD88/NF‐κB signaling pathway, leading to inflammatory responses.^[^ ^40^ ^]^ Additionally, it was reported that the specific *Tp* protein Tp17 can promote cytokine secretion through TLR4 activation.^[^ ^41^ ^]^ These results suggest that *Tp* can activate the TLR4 signaling pathway and trigger inflammatory responses through induced exosomes and specific proteins.

In addition to the brain tissue damage caused by inflammatory cells, the direct injury resulting from *Tp* infection is worthy of further investigation. Multi‐omics analyses have indicated that live spirochetes can stimulate cerebral microvascular endothelial cells, causing disruptions in extracellular matrix (ECM) regulatory proteins such as LOX and LOXL2.^[^ ^42^ ^]^ These disruptions lead to structural alterations and reduced expression of ECM components. In addition, *Tp* can degrade peri‐vascular connective tissues by secreting hyaluronidase and TP0750, and disrupt VE‐cadherin, an essential component of intercellular junctions.^[^ ^43^, ^44^ ^]^ As a result, in addition to indirect damage through inflammation and immune responses, *Tp* can directly destroy tissue structures. These findings emphasize the importance of further investigating the direct impact of *Tp* on CNS injury and the mechanisms involved and the establishment of in vitro organoid model of direct *Tp* infection is next step to study the pathogenesis of syphilis and NS.

A major limitation of this study is the small size of brain tissue samples, as it is difficult to obtain a large number of brain tissue samples from patients with NS. Thus, several biomarkers identified in our previous large‐scale CSF proteomics study, ^[^ ^17^ ^]^ such as SEMA7A, SERPINA3, and ITIH4, showed a similar expression trends but without statistical significance in brain tissues due to the sample size limitation. Multicenter and long follow‐up studies could help to further improve the correlation analysis of proteomics identification results between brain tissues and CSF from NS patients. In addition, there is a lack of investigation of the roles of specific cell types in the brain tissues of NS in this study. In‐depth exploration of the functions of different nerve and immune cells by scRNA‐seq analysis would be useful in understanding the pathogenesis of the disease. Another limitation of our brain organoids is the lack of functional vascular structures, which limits in vitro studies of the mechanisms of vascular invasion and BBB damage in NS. Further development of fully functional vascularized organoids as in vitro models of *Tp* infection is needed in the future.^[^ ^45^ ^]^

## 4 Experimental Section

### Ethics Approval and Patient Consent Statement

This study was approved by the ethics committees of Peking Union Medical College Hospital (Approval IDs: ZS‐1754 and ZS‐1105). According to the protocol approved by the institutional review boards of Peking Union Medical College Hospital, all participants provided written informed consent prior to the collection of CSF samples via lumbar puncture. All case metadata, including age, sex, and diagnostic information for NS, are provided in Table S1 (Supporting Information).

### Cerebrospinal Fluid Sample Collection

The CSF samples (n = 3) were collected and stored according to international guidelines.^[^ ^46^ ^]^ The puncture location was between lumbar spine L3/L4, L4/L5, or L5/S1, and the samples were collected into sterile polypropylene tubes, divided into 0.5 mL aliquots, and frozen at ‐80 °C within 1 h. The inclusion criteria for the NS group were positive results of the rapid plasma reagin (RPR) test and particle agglutination assay for antibodies against *Tp* (TPPA) in CSF. The inclusion criteria for the NC group were negative results of RPR and TPPA results in syphilis patients without CSF pleocytosis (>5/µL) or elevated CSF protein levels (>45 mg dL^−1^), and the absence of any characteristic signs or symptoms consistent with NS.

### Brain Tissue Sample Collection

The brain tissue samples (n = 3) were collected from paraffin‐embedded sections of the frontal and temporal lobes. The presence of spirochetes was confirmed by Warthin‐Starry staining of brain tissue samples from NS patients.^[^ ^47^ ^]^ Control brain tissue samples (n = 5) were taken from the postmortem tissues of individuals who did not have syphilis and any encephalopathy. All these samples were obtained from the Pathology Department of Peking Union Medical College Hospital.

### Culture of Brain Organoids

This protocol for brain organoid culture was modified from previous research.^[^ ^20^ ^]^ For the culture of brain organoids, hiPSCs were seeded into 96‐well round‐bottom ultralow attachment plates at a concentration of 9000 cells per well for culture in embryoid body (EB) formation medium (Essential 8 medium containing 50 µM Y‐27632). After one week, all the EBs were transferred to a 24‐well low‐attachment plate with neural induction medium (DMEM‐F12 supplemented with 1% N2, 1% GlutaMAX, 1% MEM‐NEAA and 1 µg mL^−1^ heparin). Five days later, the EBs were embedded in Matrigel droplets and transferred to low‐attachment 6‐well plates with differentiation and expansion media (a 1:1 mixture of DMEM/F12 and neurobasal media supplemented with 1:200 N2, 1:100 B27 without vitamin A, 0.1 mM 2‐mercaptoethanol, 1:4000 insulin, 1:100 GlutaMAX, and 1:200 MEM‐NEAA). After 4 days, the aggregates were transferred to a shaker containing differentiation and expansion media as above, except for B27 supplemented with vitamin A, which was used and cultured until day 40 or above.

### Isolation of PBMCs from NS Patient Blood and Coculture with Brain Organoids

The isolation of PBMCs from the blood of patients with NS was performed according to the manufacturer's protocols. After the collection of PBMCs, the brain organoids were treated with PBMCs at low (1 × 10^4^ mL^−1^) or high (1 × 10^6^ mL^−1^) concentrations or left untreated as controls. Fresh medium was added every 3–4 days. Fourteen days later, the organoids were collected and fixed.

### Protein Sample Preparation, Mass Spectrometry (MS) Analysis, and Data Processing

The paraffin‐embedded brain tissues and brain organoids were scraped into EP tubes and then washed successively with xylene, ethanol, and water to remove the paraffin. After disulfide bond reduction, the extracted sample proteins were digested with Lys C (1 µg at 37 °C for 4 h) and trypsin (enzyme‐to‐substrate ratio of 1:50) at 37 °C for 16 h, desalted using C18 cartridges, and vacuum‐dried using a SpeedVac.

The peptide fractions were measured using an Orbitrap Q Exactive HF‐X mass spectrometer (Thermo Fisher Scientific) coupled with a nanoflow LC system (EASY‐nLC 1200, Thermo Fisher Scientific). The data‐dependent acquisition (DDA) mode was used to construct a transition library, and the data‐independent acquisition (DIA) scan mode was used for all samples. DIA analysis was performed with one full MS event, followed by 42 MS/MS windows in one cycle. The full MS settings included an ion target value of 3 × 10^6^ charges in the 350–1500 m/z range, with a maximum injection time of 50 ms and a resolution of 60000 at m/z 200. The DIA precursor windows ranged from 378 m/z (lower boundary of the first window) to 1345 m/z (upper boundary of the 42nd window). The MS/MS settings included an ion target value of 1 × 10^6^ charges for the precursor window, an Xcalibur automated maximum injection time, and a resolution of 30 000 at m/z 200.

The DDA data were loaded into Spectronaut (v.14.10.201222.47784; Biognosys, Switzerland) to generate the sample‐specific spectral library. Then, the raw DIA data were processed on Spectronaut against the UniProt human database (downloaded on 2019‐7‐31, containing 73 940 proteins) using the default settings. In brief, the searches used carbamidomethylation as the fixed modification and acetylation of the protein N‐terminus and oxidation of methionines as the variable modifications. A trypsin/P proteolytic cleavage rule was used, permitting a maximum of two cleavages and a peptide length of 7–52 amino acids. Protein intensities were normalized using the “local normalization” algorithm in Spectronaut based on a local regression model; the retention time prediction type was set to dynamic iRT and correction factor for a window. The mass calibration was set to local mass calibration. Decoy generation was set to an inverse trend. Interference correction at the MS2 level was enabled by removing fragments for quantification based on the presence of interfering signals but maintaining at least three fragments for quantification. Finally, the false discovery rate was estimated using the mProphet approach and set to 1% at the peptide level.

### CSF Cell Collection, scRNA‐Seq, and scRNA‐Seq Data Analysis

During the experiment, it was found that there were several thousand cells µL^−1^ in the CSF of NS patients, while there were only dozens to hundreds of cells µL^−1^ in the CSF of control individuals. Therefore, only the CSF of NS patients was collected for single‐cell transcriptome analysis to determine the types and abundances of immune cells. The CSF samples from patients with NS (n = 3) were collected and centrifuged at 500 × *g* to generate single‐cell gene expression libraries. After three washes with cold 3% BSA solution, the cells were resuspended in 3% BSA. Cell viability and live‐cell count were determined with trypan blue staining to ensure that the final cell concentration and viability were suitable for sequencing. Then, single‐cell 3′ RNA‐seq experiments were conducted using a Chromium single‐cell system (10× Genomics) and an Illumina NovaSeq 6000 sequencer (S4 v.1.5).

Cell Ranger software (10× Genomics) (https://support.10xgenomics.com/v6.1.1) was used to process the raw scRNA‐seq data. The FASTQ files were aligned to the human genome and transcriptome (hg38) to generate a gene expression matrix. The R package Seurat (v.4.0.2) was used for further data analysis. The batch effects among three samples were first removed using the “RunHarmony” function. Next, cells with fewer than 500 or more than 4000 detected genes were filtered, and those with more than 10% mitochondrial genes were further excluded. A total of 20327 cells remained. After log‐normalization, the top 2000 highly variable genes were determined using the “FindVariableFeatures” function for downstream bioinformatics analyses. The top 20 principal components of the principal components analysis (PCA) were used to perform uniform manifold approximation and projection (UMAP) clustering. The “FindAllMarkers” function was used to identify cluster markers with the following settings: min percentage of cell‐expressed = 0.25, log fold change threshold = 0.25, and min percentage of the gene expressed = 0.1. Differentially expressed genes between two groups of cells in the scRNA‐seq data were identified using the Wilcoxon rank‐sum test. Differences for which the Benjamini‐Hochberg adjusted *p*‐value was less than 0.01 were considered to indicate statistical significance.

### Hematoxylin and Eosin (H&E) Staining, Immunohistochemistry, and Immunofluorescence

Samples were fixed in 4% formaldehyde overnight at 4 °C and then processed using a dehydration gradient. After being embedded in paraffin, the samples were cut into 4‐µm‐thick sections for H&E staining, immunohistochemistry, and immunofluorescence analysis. For H&E staining, the sections were deparaffinized and then sequentially immersed in hematoxylin, hydrochloric ethanol, 1% ammonia, and eosin. Finally, the sections were rehydrated and sealed with neutral resin before image scanning. For immunohistochemical staining, the sections were deparaffinized and heated in a microwave to a boil for at least 15 min with antigen retrieval buffer and then treated with 3% H_2_O_2_ to remove endogenous peroxidase. The sections were then blocked and stained with primary antibodies overnight at 4 °C. Afterward, the sections were stained with a secondary antibody and DAB substrate for color development. Finally, the sections were restained with hematoxylin, rehydrated, and sealed as described for H&E staining. For immunofluorescence staining, the sections were deparaffinized and heated in a microwave to a boil for at least 15 min with antigen retrieval buffer. Then, the sections were blocked and incubated with primary antibodies overnight at 4 °C. After incubation with secondary antibodies and counterstaining with DAPI, the sections were sealed with Fluoro‐Gel for imaging. Images were taken at 10×/20×/40×/60× magnification by using a Nikon A1R+N‐STORM confocal microscope.

### Statistical and Bioinformatics Analysis

R statistical software v.4.3.2 was used to conduct all analyses and visualization. The quantified protein expression values were log2 transformed and mean‐centered. Pairwise comparisons to determine the proteins whose expression significantly differed in brain tissue samples between the NS (n = 3) and control (n = 5) groups and in brain organoids between the PBMC^low^ (n = 3), PBMC^high^ (n = 3), and control (n = 3) groups were performed by a moderated *t test* using the R package limma (version 3.58.1). The significantly differentially expressed proteins were determined according to the Benjamin–Hochberg (BH) adjusted *p*‐value < 0.01. Differentially expressed proteins with log2‐fold changes > 1 or < ‐1 were considered significantly upregulated or downregulated, respectively. Coexpression analysis revealed a gradual increase and gradual decrease in the expression levels of proteins identified in brain organoids among control, PBMC^low^, and PBMC^high^ samples. Differences according to the BH‐adjusted *p‐value* < 0.01 for both the PBMC^low^ versus Control and PBMC^high^ versus Control were considered statistically significant, and the proteins with log_2_ (PBMC^low^/Control) > 0 and log_2_ (PBMC^high^/PBMC^low^) > 0 were identified as gradually increasing, while the proteins with log_2_ (PBMC^low^/Control) < 0 and log_2_ (PBMC^high^/PBMC^low^) < 0 were identified as gradually decreasing.

The online tool DAVID (https://david.ncifcrf.gov/)^[^ ^48^ ^]^ was used to annotate the proteins according to biological processes and molecular functions via Gene Ontology^[^ ^49^ ^]^ analysis. The enrichment of biological pathways was analyzed via the Kyoto Encyclopedia of Genes and Genomes^[^ ^50^ ^]^ pathway. The protein interactome network was constructed using Cytoscape (v.3.8.2),^[^ ^51^ ^]^ and the protein‐protein interactions were retrieved from the STRING database.^[^ ^52^ ^]^ PCoA of the proteins whose values in each sample were deemed valid was performed using the R package ape.^[^ ^53^ ^]^ Volcano plots and heatmaps for the quantified values for proteins whose expression significantly differed between groups were produced using the R packages ggplot2 and ComplexHeatmap^[^ ^54^ ^]^ (distance: Pearson; linkage: complete).

## Conflict of Interest

The authors declare no conflict of interest.

## Author Contributions

Q.Z., J.M., J.Z., H.Z., and M.L. contributed equally to this work. L.L. conceived the overall study and designed experiments. L.L. and J.L. had full access to all the data in the study and took responsibility for the data analysis accuracy. H.Z., J.L., J.Z., H.Z., H.G., and Y.W. performed the CSF and brain tissue samples preparation and clinical information collection. J.M., M.L., and L.L. performed proteomics and scRNA experiments and bioinformatics analysis. Q.Z. contributed to the brain organoids culture. Q.Z., J.Z., H.Z., and H.G. performed most biological and functional experiments. L.L., Q.Z., and J.M. wrote and edited the manuscript. L.L. and J.L. provided funding support. All authors made important comments to the manuscript.

## Supporting information



Supporting Information

Supplemental Table 1

Supplemental Table 2

Supplemental Table 3

Supplemental Table 4

Supplemental Table 5

## Data Availability

All proteomics experiment data generated in this study have been deposited to the ProteomeXchange Consortium via iProX^[^ ^55^, ^56^ ^]^ repository with identifier PXD053294. The equipments, reagents, and supplies are available in the Table S5 (Supporting Information).
