# Supplementary material for: A Study on the Inflammatory Response of the Brain in Neurosyphilis
Source: Adv Sci (Weinh). 2024 Nov 22;12(5):2406971. doi: 10.1002/advs.202406971 (PMC11792053; doi:10.1002/advs.202406971)
Supplement: Supplementary file 1 — Supporting Information [file ADVS-12-2406971-s005.docx]

Supporting Information

**A Study on the Inflammatory Response of the Brain in Neurosyphilis**

*Qiyu Zhang^†^, Jie Ma^†^, Jia Zhou^†^, Hanlin Zhang^†^, Mansheng Li^†^, Huizi Gong, Yujie Wan^1^, Hengyi Zheng^*^, Jun Li^*^, Ling Leng^*^*

^†^ These authors contributed equally to this work.

* Correspondence: Hengyi Zheng (zhenghy62@hotmail.com) or Jun Li (lijun35@hotmail.com) or Ling Leng (lengling@pumch.cn or zhenlinger@126.com)

**Supplementary Figures**


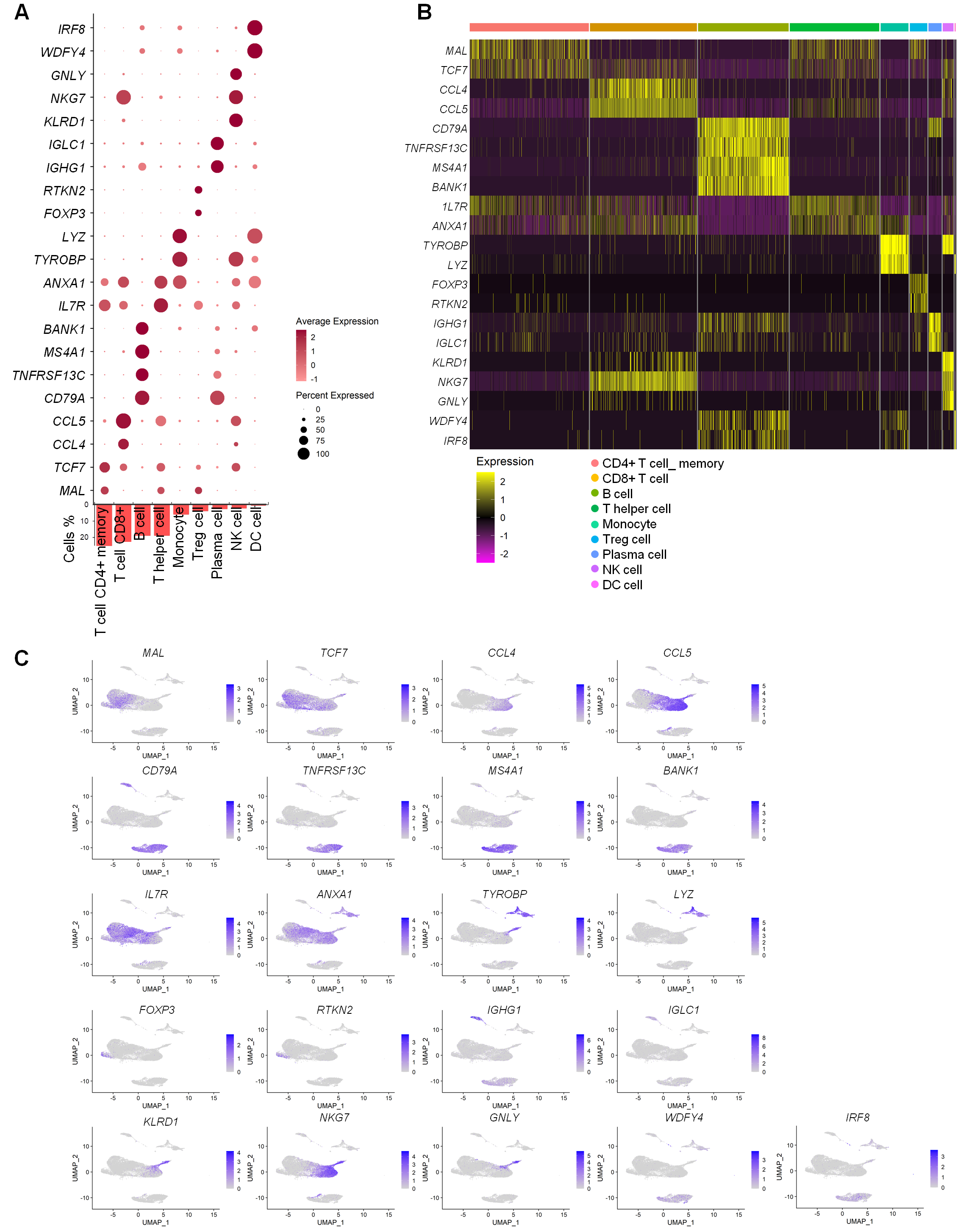


**Figure S1.** scRNA-seq gene expression signatures for different cell subtypes in the CSF from NS patients. (A) Expression of key gene markers and the percentage distribution of different cell clusters. (B) Heatmap of the normalized expression of the top differentially expressed genes per cell cluster in the CSF of NS patients (n = 3). (C) UMAP plots of the specific marker genes for cell subtype classification.

**
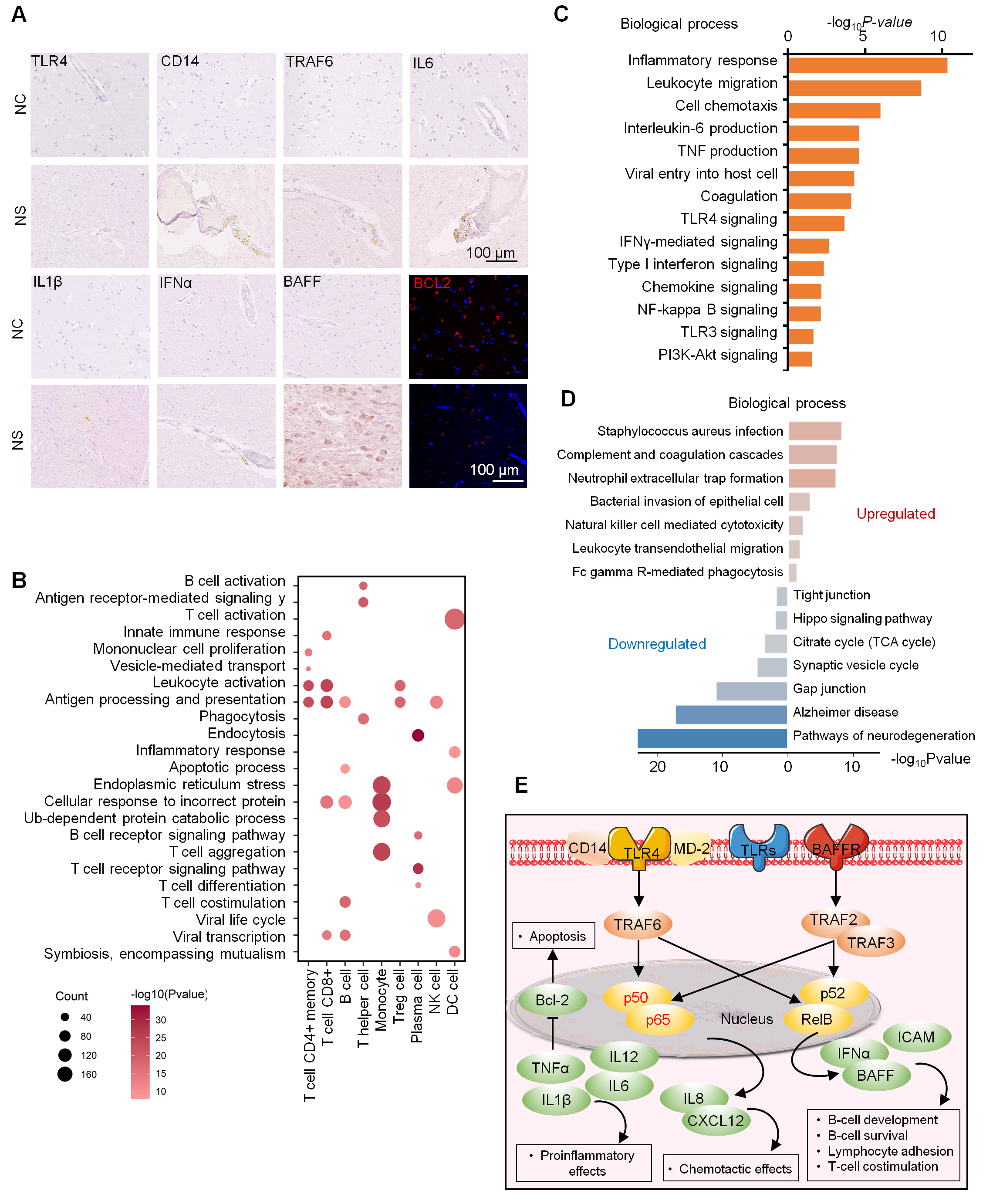
**

**Figure S2.** Functional characteristics of different cell subtypes in the CSF of NS patients. (A) Histological staining analysis of inflammatory pathway- associated proteins (TRL4, CD14, TRAF6, IL6, IL1β, IFNα, and BAFF) and an antiapoptotic protein (BCL2) (scale bar: 100 μm) in the brain tissues of NS patients (n = 3). (B) Biological process enrichment analysis of the differentially expressed genes in different cell subtypes in the CSF of NS patients (n = 3). Differentially expressed genes between two groups of cells in the scRNA-seq data were identified using the Wilcoxon rank-sum test. Differences for which the BH adjusted *p* value was less than 0.01 were considered to indicate statistical significance. The red gradient column represents the degree of enrichment of biological processes according to -log10 (P value). The circle size represents the number of genes enriched in a biological process. (C) Biological process enrichment analysis of the differentially expressed genes of the subsets of macrophages in the CSF of NS patients. (D) Biological process enrichment analysis of differentially expressed proteins in the brain tissues from NS patients (n = 3) compared to control individuals (n = 3). (E) Mechanistic diagram of immune process-associated pathway activation in NS patient brain tissue. The diagram shows that the Toll-like receptor signaling pathway mediates the classical and nonclassical NF-κB signaling pathways and the downstream production of inflammatory factors to induce an inflammatory response, immune cell activation, and apoptosis.

**
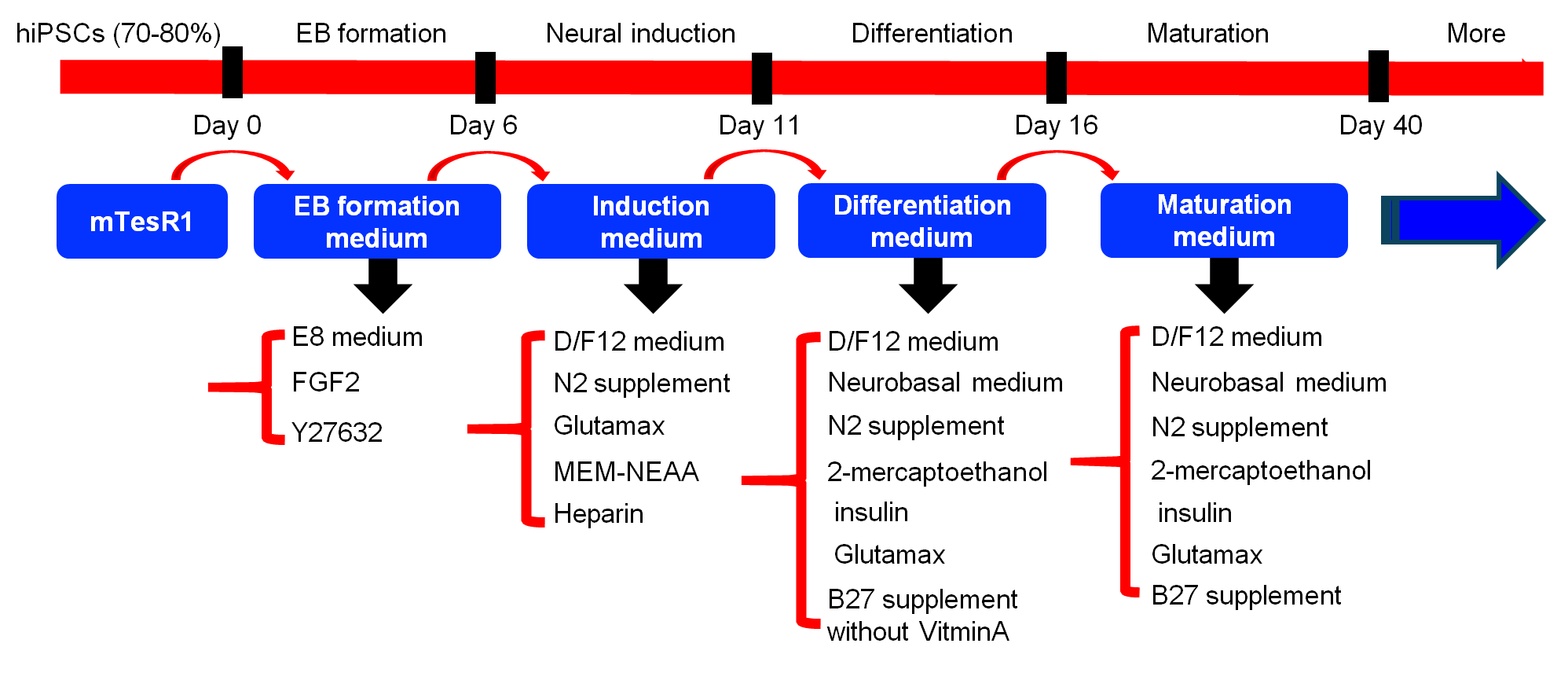
**

**Figure S****3.** Differentiation protocol of hiPSC-derived brain organoids.

**
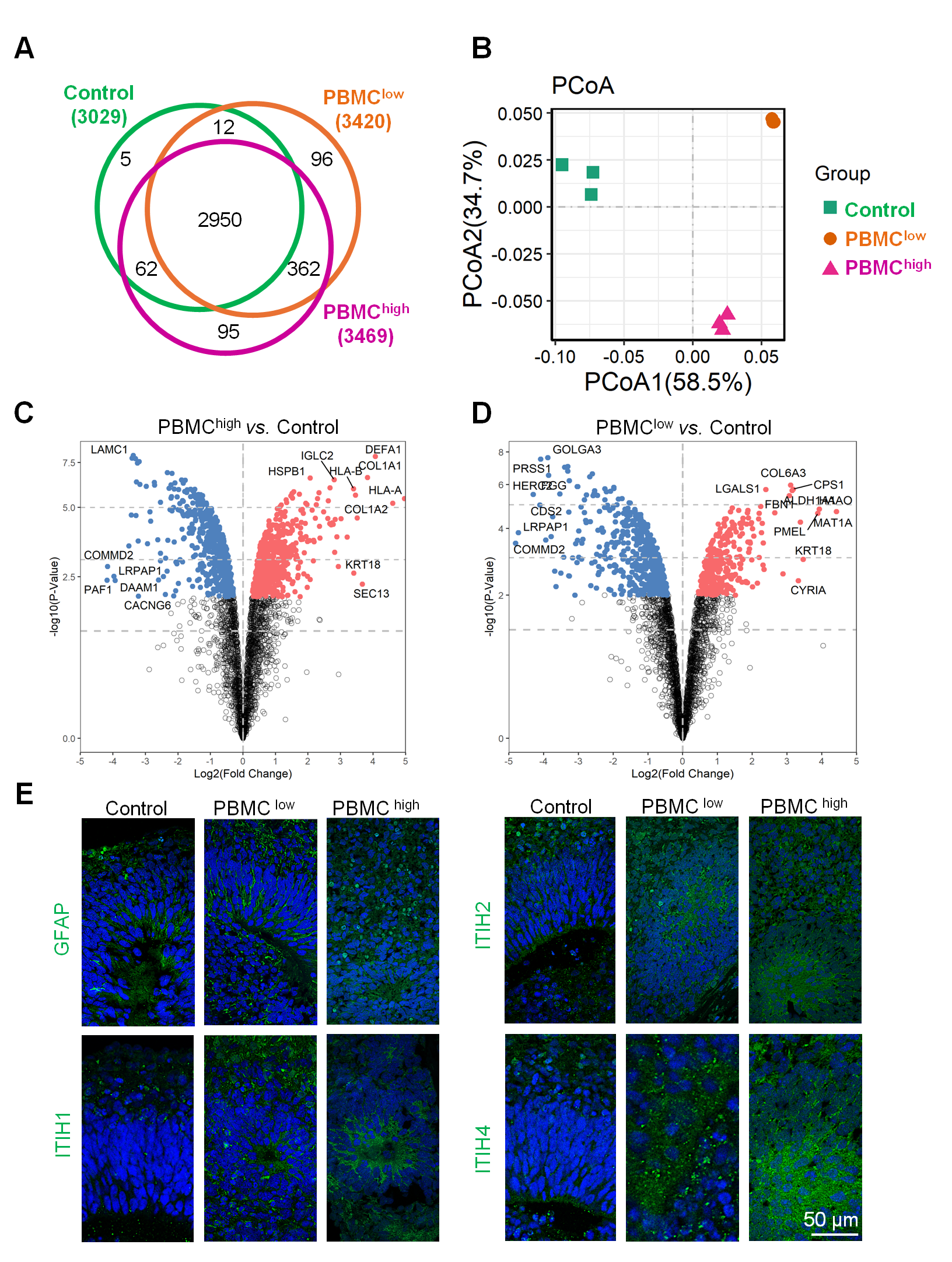
**

**Figure S4.** Proteome profile of human brain organoids cocultured with PBMCs. (A) Venn diagram of proteins identified in brain organoids cocultured with different concentrations (0, low, or high) of PBMCs. (B) PCoA of the proteome profiles of the PBMC^low^ (n = 3), PBMC^high^ (n = 3), and control (n = 3) groups. Green squares, orange circles, and pink triangles represent the control, PBMC^low^ and PBMC^high^ groups, respectively. Volcano plots of–log_10_ p value vs. log_2_ protein abundance comparisons for PBMC-cocultured organoids between the PBMC^high^ (C), PBMC^low^(D) and control groups. Differential expression analysis is performed using a moderated t test implemented in the R package limma. Proteins outside the significance threshold lines (−log_10_ (p value) > 2 and |log_2_ (PBMC^low^ or PBMC^high^/control| > 1) are considered statistically significant and shown in red (upregulated) or blue (downregulated). (E) Immunofluorescence analysis of GFAP, ITIH1, ITIH2, and ITIH4 in brain organoids cocultured with PBMCs (scale bar: 50 μm).
